# Dissecting Deep Learning Networks—Visualizing Mutual Information

**DOI:** 10.3390/e20110823

**Published:** 2018-10-26

**Authors:** Hui Fang, Victoria Wang, Motonori Yamaguchi

**Affiliations:** 1 Computer Science Department, Liverpool John Moores University, Liverpool L3 3AF, UK; 2 Institute for Criminal Justice Studies, University of Portsmouth, Portsmouth PO1 2HY, UK; 3 Department of Psychology, Edge Hill University, Ormskirk L39 4QP, UK

**Keywords:** deep learning, convolutional neural networks, information theory, mutual information, visualization

## Abstract

Deep Learning (DL) networks are recent revolutionary developments in artificial intelligence research. Typical networks are stacked by groups of layers that are further composed of many convolutional kernels or neurons. In network design, many hyper-parameters need to be defined heuristically before training in order to achieve high cross-validation accuracies. However, accuracy evaluation from the output layer alone is not sufficient to specify the roles of the hidden units in associated networks. This results in a significant knowledge gap between DL’s wider applications and its limited theoretical understanding. To narrow the knowledge gap, our study explores visualization techniques to illustrate the mutual information (MI) in DL networks. The MI is a theoretical measurement, reflecting the relationship between two sets of random variables even if their relationship is highly non-linear and hidden in high-dimensional data. Our study aims to understand the roles of DL units in classification performance of the networks. Via a series of experiments using several popular DL networks, it shows that the visualization of MI and its change patterns between the input/output with the hidden layers and basic units can facilitate a better understanding of these DL units’ roles. Our investigation on network convergence suggests a more objective manner to potentially evaluate DL networks. Furthermore, the visualization provides a useful tool to gain insights into the network performance, and thus to potentially facilitate the design of better network architectures by identifying redundancy and less-effective network units.

## 1. Introduction

Deep Learning (DL) is a powerful neural network technique, which has been one of the most revolutionary developments in artificial intelligence (AI) research in this decade, e.g., [1,2,3,4,5,6]. With the aid of graphical processing unit (GPU), DL has been used in many domains that are difficult for conventional machine learning (ML) methods to manage. These include image classification and analysis [1,4], natural language processing [5], speech recognition [2], and playing complex games [3,6]. These DL models are capable of learning either discriminative or generative representations, from a large amount of training data, via the stochastic gradient descent (SGD) method. A DL network normally contains millions of trainable parameters and it is not an easy task to design such a large network. Before training a network, there are numerous hyper-parameters to be defined heuristically. These include number of layers, number of convolutional kernels, kernel size, stride length, selection of activation functions and epochs for convergence. However, there is not a standard guideline or metrics to guide the selection of appropriate hyper-parameters in order to achieve an optimistic performance.

In cross-validation evaluation, the accuracy of model outputs provides an objective score to compare the performance of DL networks. However, the accuracy rate does not provide insights into the networks. For example, it is not traceable to know which components of a network are responsible for the effectiveness of the network, or what roles a given unit plays in the network. Consequently, the DL networks are still treated as “magic classifiers” that mysteriously tackle hard tasks that conventional ML cannot solve. The lack of an in-depth theoretical understanding of DL limits further applications of the technique in various areas, such as network security and digital healthcare.

The present study aims to provide a microscopic view of the DL networks based on the information theory. Our focus is on mutual information (MI)—a metric quantifying the amount of information shared between two random variables. The MI is a theoretic measurement, which is independent of any classifiers to reflect the relationship between the variables even if the relationship is highly non-linear and hidden in high-dimensional data. There are two key advantages of introducing MI as an indicator in an evaluation framework. First, when two networks achieve similar accuracy rates, the one with a higher MI is more suitable for the classification tasks with multi-instance decision making. For example, in Reference [7], video sequences are required to classify into high-level human actions by considering multi-frames. A DL network with high MI will be able to perform better classification comparing to another DL network with low MI due to the well distributed feature representations of individuals in the model. Secondly, MI is able to provide an important metric to better understand DL networks. Measuring MI between DL network units, such as neurons, kernels or layers with output classification, is capable of evaluating the networks in a more detailed, objective and independent manner.

Currently, there is a growing interest in using MI to interpret and improve DL networks [8,9,10,11,12]. For instances, Reference [10] designed a dynamic learning rate for better convergence based on the MI; Reference [11] added a shortcut in an MLP network to improve the learning efficiency of the network; and Reference [12] used mutual information neural estimation (MINE) to explicitly maximize the mutual information between input data and learned high-level representations. Inspired by recent information plane work [8,9], we visualize the mutual information between layers/kernels in several popular DL networks in order to gain a better theoretical understanding of these networks.

In our paper, we evaluate our framework by deploying three networks, including multi-layer perceptron (MLP) network [13], Le-Net network [14], and DenseNet [15]. Multi-layer perceptron is a traditional fully connected neural network. It has been set as a standard DL network—a benchmark ML research. From 2012, convolutional neural networks have achieved significantly better results compared to the fully connected network architecture. Le-Net, as the most classical convolutional network (CNN), has been re-designed, i.e., the tanh activation function is taken place by a rectified linear unit (ReLU) activation function, to be the most popular CNN network benchmark. DenseNet is a newly designed networks with direct connections between each layer. The network practically alleviates the vanishing-gradient problem, encourages feature reuse and controls the number of parameters well. In this study, it is desired to achieve an in-depth understanding of the networks.

In comparison with the work in [8,9,10,11,12], the contributions of our study can be highlighted as follows: (i) we extend the information plane analysis from fully connected neural networks to the convolutional neural networks that include the LeNet and the DenseNet. Whilst in most of the references, the results are only demonstrated by using MLP networks; (ii) instead of only calculating the MI between layers, we consider convolutional kernels as individual units and visualize the MI between the kernels and the output labels in order to illustrate the roles and the evolutional patterns of the kernels during training; (iii) we show that the MI is used to guide the visualization of response maps extracted from convolutional kernels with visual inspection, which helps to identify redundancy and less effective kernels and allows better transfer of learning; and (iv) we demonstrate that the MI visualization tools can be used to select effective hyper-parameters, such as layer structure, stride length, and number of epochs.

This paper is structured as follows: Section 2 presents a review of relevant techniques from the existing literature, including deep learning models, information theory and deep learning visualization methods. Section 3 describes the details of the deep learning networks used in our work. Section 4 explains how to estimate the MI and use the information plane to analyze the DL networks. Section 5 visualizes MI and its evolutional patterns from the convolutional blocks in the CNNs. Section 6 summarizes main insights obtained from the visualization techniques applied to the CNNs in the present study, and Section 7 draws a conclusion and suggests the future works.

## 2. Related Work

### 2.1. Deep Learning Networks

Classical convolutional networks are widely used in the pattern recognition community in recent years. AlexNet is an eight-layer network, which includes five convolutional layers and three fully connected layers, to classify millions of images into 1000 object categories. The use of CNNs has been promoted heavily and adapted in classification applications since the work achieved the best performance in the ImageNet ILSVRC 2010 contest [1]. Some examples of the applications include: Google applied a CNN to create a facial recognition tool, named FaceNet [16]; Simonyan et al. [17] and Fang et al. [7] proposed two-stream CNN networks, named spatial and temporal nets, to integrate appearance and motion features in order to recognize high-level human actions from video sequences; Google DeepMind built a 13-layer policy CNN to evaluate board positions of the Go game in order to reduce the sampling in the searching tree [6].

Additionally, DL networks have also been applied to image synthesis and style transfer in computer graphics. For instance, Reference [18] presented a fusion layer to merge local feature with global priors that are both extracted from CNNs to colorize greyscale images. Since the invention of Generative Adversarial Networks (GAN) [19], optimizing a generator network and a discriminative network simultaneously, has become a standard method for image synthesis to generate more realistic images. Radford et al. further developed Deep Convolutional Generative Adversarial Networks (DCGANs) to improve the performance of unsupervised learning algorithms by using the CNN architecture in the GAN networks [20].

Recently, the development trend of DL is that the networks either become much deeper, (e.g., Highway Networks [21] and ResNets [22]), or start reusing convolutional kernels from previous layers [15]. However, further increase of layers becomes riskier when the combination of layers is treated as a black box tool. As a result, a better understanding of the network blocks, layers, kernels, and neurons will be able to help the redundancy reduction, network simplification and potential overfitting issues identification in order to make the networks more flexible for transfer learning.

### 2.2. Information Theory

One way to gain a better understanding of the functions of hidden layers is to reveal the relationships between hidden layers and the inputs/outputs of models. Information theory provides an objective way to quantify the relationships among two random variables by analyzing the uncertainty of the random variables (feature vectors). Entropy, conditional entropy and MI, as a set of objective metrics, have been successfully adapted into many fields. For example, MI is one of the most common metrics in medical image registration [23]. A pair of images from various sources or modalities can be aligned by maximizing the MI between them. In [24], MI was maximized in a GAN model to obtain more disentangled representations of handwriting digits. Thus, each dimension of the feature vector can be interpreted by observable factors, such as stroke width or leaning direction.

Information theory has also received much attention in the field of visualization over the decade [25,26,27,28]. Chen et al. proposed a theoretical framework to examine whether information theory can be used to quantify the visual information [25]. Xu et al. designed a streamline rendering method via introducing the entropy field in flow visualization [26]. Alsakran et al. proposed to use entropy-related measures to facilitate more effective categorical data visualization when processing high dimensional data, e.g., sorting categories, optimizing order of multiple coordinates or arranging coordinate space [28]. A survey [27] summarized a variety of applications that use the information theory in visualization applications.

### 2.3. Deep Learning Analysis via Visualization

Recent studies investigated visualization techniques as an intuitive way to gain the insight of DL methods, e.g., [8,29,30,31]. Zeiler et al. [29] visualized the most discriminant features in images via a Deconvnet operation. The operation projects the most activated feature maps back to the original image space and it facilitates the further tuning of the network. In [30], Bau et al. proposed a framework called Network Dissection (ND) to evaluate the interpretative power of individual hidden units for a set of semantic concepts. Network dissection compared the overlapped regions between the spatial locations activated by individual convolutional units and the ground truth labels of visual concepts from the Broden dataset [30] in which there are many high semantic labels to interpret the disentangled representation of the units. In [31], Liu et al. presented a visual analytics system to diagnose and refine CNNs by clustering neurons and visualizing activation maps.

The information plane work [8,9] has potential to provide a theoretical basis for DL network analysis. In Reference [8], it is suggested that two stages, optimization and compression of the information, occur over the iterations to make DL networks outperform other models. In our study, we extend the information theoretic approach in two aspects. First, we investigate the MI changes in several popular DL networks to analyze their evolutional pattern; second, we further visualize the MI between representations extracted from individual kernels in different hidden layers and the output classification during the evolution of the CNN in order to gain a better understanding of the basic computational units of the networks. We apply several visualization techniques, including statistics visualization, pixel visualization and heat-map, to summarize MI dynamic patterns. These techniques allow interpretations of the DL networks in an information theoretic language.

## 3. Deep Learning Networks

Deep learning networks consist of many stacked layers to pass raw input data into a sequence of transformations to maximize the final classification accuracy. The layers, as one type of the basic units of deep learning networks, include convolution layers, activation layers, pooling layers, normalization layers, dropout layers and fully connected layers. Among these layers, the convolutional layer and fully connected layer are the core components in DL networks. Convolutional layer refers to groups of convolving kernels used to find distinctive local features to support the classification task. In traditional image processing pipeline, convolutional kernels are handcrafted to obtain response maps from the original image as the feature extraction step. For examples, the Sobel kernel or Prewitt kernel are used to extract edge information, whereas a Gaussian smoothing kernel is used to get a noise-reduced blurred image. In the CNN pipelines, the convolution kernels are initialized randomly and evolved to learn saliency in order to improve the ultimate classification performance. Stacks of feature maps are calculated by convolution operations across layers and kernels. As shown in the Equation (1), the response map of the *i* layer convolving from the *j* kernel is obtained by weighting and summing the local input signals from the *C* channels and spatial window whose size is (*H*, *W*).

(1) $$fm_{i,j}\left(x,y\right)=I_{i}*K_{i,j}=\overset{C}{\underset{c=0}{\sum }}\overset{\frac{H}{2}}{\underset{u=-\frac{H}{2}}{\sum }}\overset{\frac{W}{2}}{\underset{v=-\frac{W}{2}}{\sum }}I_{i}\left(c,x+u,y+v\right)k_{i,j}\left(c,u,v\right)$$

where the *x* and *y* represent the spatial coordinate of the feature map; *I* is either the original image or a feature map from previous convolutional layer; *C* is the channel number and k are the convolutional kernels. In contrast, fully connected layer refers to a number of neurons that are connected with all the activations passed from a previous layer to obtain global features. This type of dense connection is the basics of the traditional multilayer perceptron (MLP) neural networks [32].

There are many other non-trivial layers existing between two convolutional layers, two fully connected layers or between these two types of layers. These layers include activation layers, pooling layers, normalization layers and dropout layers. The normalization and activation layers work as linear or non-linear mapping layer to normalize or filter the active features by using normalization or activation functions, such as Sigmoid function, Tanh function, or ReLU function. The pooling layers are added into CNNs to down-sample the activation maps from previous layer when preserving the average or maximum local active response values. Dropout layers are designed to drop out some neurons during training in order to minimize overfitting.

**Figure 1:**
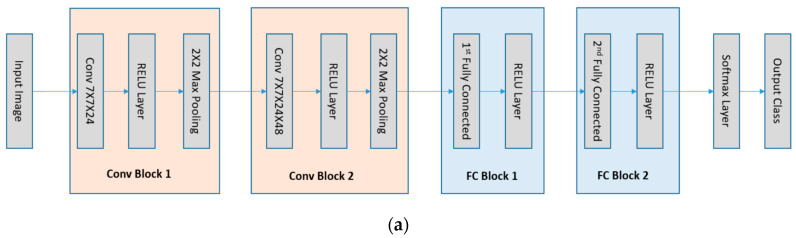

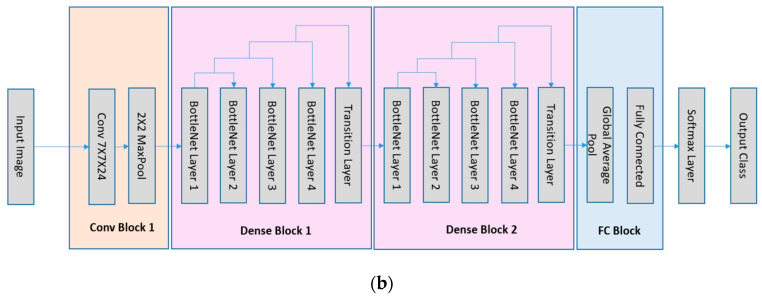
The LeNet5 and the DenseNet architectures used in the study. The MI values of kernels after each block are calculated for analysis and comparison. (**a**) The LeNet5 contains 2 convolutional blocks and 2 fully connected blocks; (**b**) the DenseNet contains 1 convolutional block, 2 dense blocks and 1 fully connected block.

**Figure 2:**
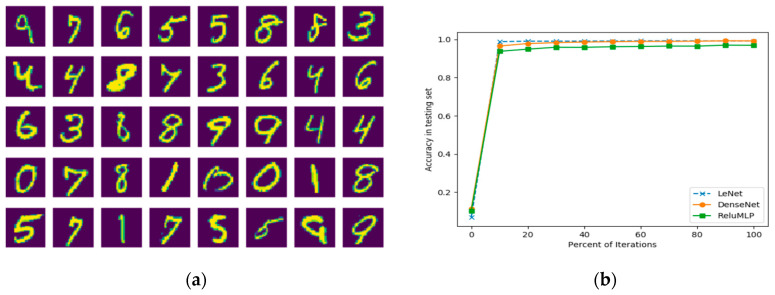
The classification accuracy is unable to provide insight of the networks: (**a**) some examples in the MNIST dataset; (**b**) classification accuracy of 3 DL networks on the test set of MNIST.

The network architectures in our investigation are the LeNet5 [14] and the DenseNet [15] that are the convolutional neural networks (see Figure 1). As CNNs become much deeper than the traditional neural networks, the layers can be further grouped into manageable blocks. As illustrated in Figure 1a, the LeNet5 has four blocks: one 7×7×24 with stride 1 convolutional layer followed by a ReLU layer and a max-pooling layer, one 7×7×48 with stride 1 convolutional layer followed by one ReLU layer and one max-pooling layer, one 1024 dimensional fully connected layer followed by a ReLU layer, and one 10 dimensional fully connected layer. The DenseNet, as illustrated in Figure 1b, has one convolutional block, two dense blocks and a fully connected block. The first convolutional block has 7×7×24 convolutional kernels with stride 2 and a 2×2 max pooling layer. The dense block is composed of the concatenation feature map of 4 BottleNet Layers passing to a transition layer. A BottleNet layer includes a ReLU+1×1×48 with stride 1 Convolutional layer and a ReLU+3×3×12 with stride 1 Convolutional layer. The transition layer is composed of one ReLU layer, one 1×1×12 with stride 1 convolutional kernels and one 2×2 average pooling layer. The last block of the network is composed of one global average pooling layer and a 10 dimension fully connected layer. A Softmax operation is set to obtain the final classification in all the networks. Another fully connected network, ReLU-MLP [32], is also used as a benchmark for comparison. The ReLU-MLP is a multilayer perception network with a deep structure (5 fully connected layers, the numbers of neurons for the layers are 784-1024-256-128-10) and ReLU activation layers between the fully-connected layers. When evaluating the performance of DL networks, cross validation is the standard way to compare methods and algorithms. As illustrated in Figure 2, the performance of the three DL networks is compared by using the MNIST dataset [33]. This dataset is a standard collection designed for training automatic digit handwriting recognition task. It consists of 55,000 training images, 10,000 testing images, and includes handwritten digits from 0 to 9. Each image has a patch size of 28 by 28 pixels. The three networks are trained via ADAM optimizer [34], using the MNIST training set. After 50,000 iterations, the accuracy rates of the networks are 96.93%, 99.22%, and 99.23%, respectively, for ReLU-MLP, LeNet, and DenseNet (see Figure 2b). Thus, the two convolutional networks outperformed ReLU-MLP by over 2 percent. Other than that, there is little insight into the underlying functions of the networks from the accuracy plot. It is difficult to know how each basic unit of these networks contributes to the final result. Therefore, another metric is needed to answer some questions, such as how the weights in layers and kernels are evolved during training; what response features are activated from the convolutional layers and whether there are redundancy and overfitting issues in these layers.

## 4. Mutual Information Estimation and Information Plane for DL Network Analysis

MI is an objective measurement to evaluate the dependency between two random variables by quantifying the total information shared between them. It is a favorable measurement for several reasons: (i) it provides a meaningful indicator to analyze the non-linear relationship between the variables even if the patterns are hidden in higher-order statistics; (ii) it offers a measurement that is independent of any classifiers; (iii) it helps to understand the feature representations of each layer in the DL networks when the classification accuracy is impossible to detect the change of the learned features; and most importantly, (iv) MI values converge to tight error bounds to reflect the true relatedness. In this study, we take a new perspective to analyze CNN networks by visualizing the MI in the training and the convergence stage in order to understand the roles and changes of the layers in the network.

The information theory simply requires the probability distributions of two random variables, *p*(*x*) and *p*(*y*), and their joint distribution *p*(*x*, *y*). The MI can be calculated from the follow Equation:

(2) MI(X;Y)=−∫p(x)logp(x)dx−∫p(y)logp(y)dy+∫p(x,y)logp(x,y)dxdy

However, there are many different methods to estimate MI values. In real applications, different assumptions are made to estimate the entropy, joint entropy and mutual information. The most popular estimators include bin-based estimator [25], K-nearest neighbor estimator [35] and kernel-density based estimator (KDE) [36]. Binning methods are the most straightforward approaches, but they are not reliable on generalized MI estimation due to the sensitiveness of bin size which causes divergence of MI for finite sample number. K-nearest neighbor estimator in [35] is a popular non-parametric method to calculate the MI. The method defines a space that contains k nearest neighbors of the joint random variable *Z* = (*X*, *Y*), which are projected to the *X* subspace and the *Y* subspace to estimate the probability distributions.

To make our work consistent with [9,36], the KDE method in Reference [36] is used to estimate the entropy so that the MI can be calculated as follows:

(3) $$MI\left(X;Y\right)={\hat{H}}_{KDE}\left(X\right)-{\hat{H}}_{KDE}\left(X|Y\right)$$

Assuming that the variable *X* obeys a mixture of Gaussians distribution, using Kullback–Leibler (KL) divergence as the pairwise distance provides an upper bound of the mixture entropy. The kernel density estimator is simply the KL estimator with a dimensional correction, which is given by:

(4) $${\hat{H}}_{KL}\left(X\right)=\frac{d}{2}+{\hat{H}}_{KDE}\left(X\right)=\frac{d}{2}-\underset{i}{\sum }c_{i}ln\underset{j}{\sum }c_{j}p_{j}\left({µ}_{i}\right)$$

where $p_{j}\left({µ}_{i}\right)$ is the mixture probability of the component mean µ*_i_* and $c_{i}$ are the weights of component *i*. The conditional entropy can be calculated as:

(5) $${\hat{H}}_{KDE}\left(X|Y\right)=\underset{y}{\sum }w_{Y=y}*{\hat{H}}_{KDE}\left(X_{Y=y}\right)$$

**Figure 3:**
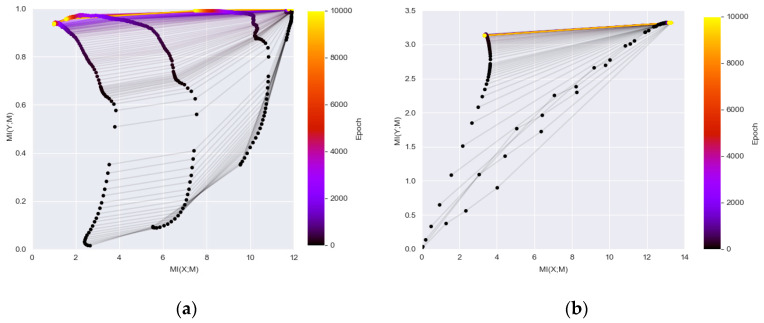
The information plane provides insight of the network dynamics: (**a**) information plane dynamics of a Tahn fully connected 12-10-7-5-4-3-2 network (as the final label has 2 classes, the maximum MI on *Y*-axis is 1 and the maximum MI on *X*-axis corresponding to the maximum possible MI between the input and hidden layers); (**b**) information plane dynamics of a ReLU fully connected 784-1024-20-20-20-10 MNIST network (as the final label has 10 classes, the maximum MI on *Y*-axis is log_2_ (10) and the maximum MI on *X*-axis corresponding to the maximum possible MI between the input and hidden layers).

Recently, there is an innovative idea to study the dynamics of DL networks via the information theory by plotting the estimated MI between the hidden layers with the input and the output during the optimization process [8,9]. The information plane plots visualize the characteristics of encoder and decoder information by calculating MI (*X*; *M_i_*) and MI (*M_i_*; *Y*) where *X* is the input variable, *Y* is the output labels, and *M_i_* represents the variables of the *i* hidden layer. In [9], two DL networks with fully connected layers are used. One network consisted of seven fully connected layers where neuron numbers are 12-10-7-5-4-3-2 respectively, and the other network was an MLP network for MNIST where neuron numbers are 784-1024-20-20-20-10 respectively (the input is transforming a 28 × 28 image patch into 784 dimensional vector). Figure 3 duplicated their results which show MI (*X*; *M_i_*) and MI (*M_i_*; *Y*) during training of the two networks. The visualization of the information plane illustrates a consistent increase of the MI values between the hidden representations of each layers and the output. Note that the leftmost curve represents the MI value change of the last hidden layer preceding the output layer and the rightmost curve is the value changes of the first hidden layer following the input layer.

The information plan visualization is built upon the information bottleneck theory (IB) [37], which is a computational framework to find minimal sufficient statistics to compress original signal as well as predict the outcomes. The plots in Figure 3 demonstrated that the goal of DL networks is to optimize the information bottleneck for each layer. Although the initialization of one type of network (e.g., LeNet) may be randomized, the evolution in the training stage has its characteristic pattern and the convergence is close to the theoretical bound of the IB. The plot is informative as it showed that the DL networks can be generalized as learning efficient representation by going through diffusion and compression phase. As a result, the compression phase and convergence to the IB bound from the layers can be used to explain the success of DL methods.

## 5. MI Visualization of Deep Learning Models

In this study, a visualization framework is designed to illustrate the MI generic and evolutional patterns in a hierarchical structure. There are three levels of illustrations: at the highest level, the information plane visualization technique is explored to show MI changes of entire blocks of the three networks; in the middle level, statistical visualization and response map visualization are used to get the MI information of the individual kernels in each block of the CNN networks; and in the bottom level, heat-map visualization is used to investigate the much detailed relationship between kernels and each class. In our proposed framework, the high-level visualization illustrates a general perspective of the model suitability to a dataset objectively while the low-level visualization shows how the models work in detailed tasks (e.g., which classes have higher correlation with the extracted representations and which do not). The three-level MI visualization is not only able to complement one another to provide a complete picture about the objective evaluation of the DL network performance but also interpret the models in order to achieve an in-depth understanding of their structures.

### 5.1. Information Plane Visualization

We investigate the information theoretical approach to comparing and analyzing different network architectures. MI has a long history to be used as a key metric to design classifiers. For example, as one of the most classical classifiers, the decision tree algorithm [38,39] makes classification by selecting the most effective features in orders via maximizing the entropy gain which is one form of MI. The weakness of the method is that its assumption about the independency between features ignores the most important higher order relationship when dealing with the high dimensional variable. As a result, it prevents the classifier from achieving high accuracy performances in more complex tasks, though MI is still a powerful gauge to measure the learned relationship. DL networks are able to learn this type of relationship by stacking many non-linear mapping functions. While the network architectures are set by designers heuristically, slight changes of a network architecture may lead to different convergence results. The information plane visualization provides a tool to objectively evaluate the convergence of the network architectures.

**Figure 4:**
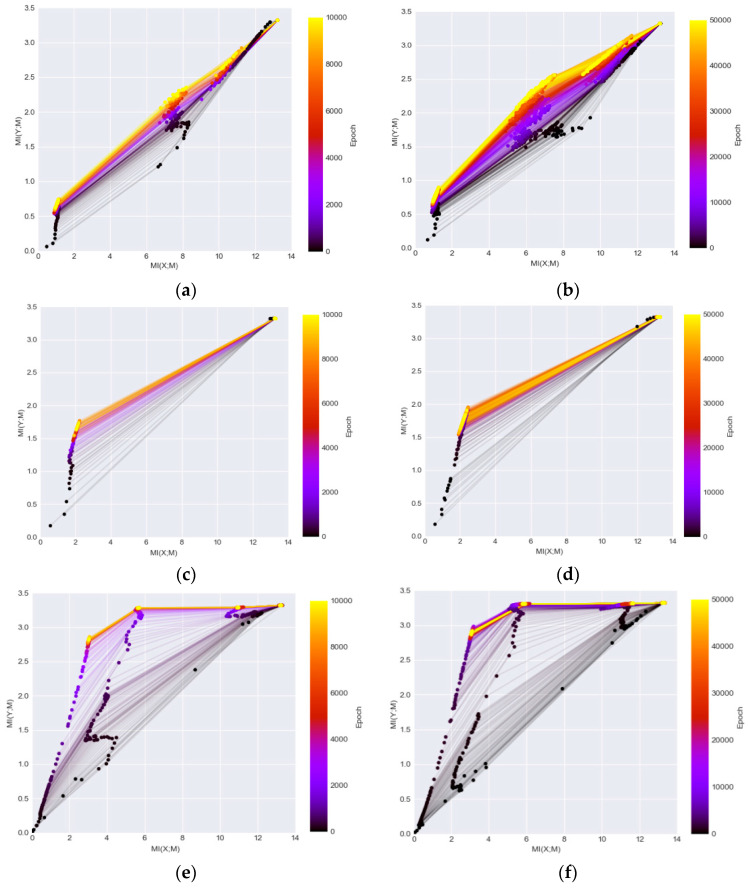
The information plane provides insight of the network dynamics (as the final label has 10 classes, the maximum MI on *Y* axis is log_2_ (10) and the maximum MI on *X* axis corresponding to the maximum possible MI between the input and hidden layers) (**a**) ReLU-MLP with 10,000 iterations; (**b**) ReLU-MLP with 50,000 iterations; (**c**) LeNet with 10,000 iterations; (**d**) LeNet with 50,000 iterations; (**e**) DenseNet with 10,000 iterations; (**f**) DenseNet with 50,000 iterations.

Figure 4 illustrates the information planes of the three DL networks. The left column shows the MI changes over 10,000 iterations and the right column shows the MI changes over 50,000 iterations. The figure demonstrates clear patterns how MI values of individual layers change in each network during the training. After the transformation of data through the networks, the final converged MI value in the last layer of DenseNet (Figure 4e,f) is higher than the MI values in the corresponding layers in the other two networks (Figure 4a–d). The higher MI values indicate that the representations extracted from the network are more informative to interpret the classes. The observation confirms the findings that the DenseNet outperforms both the LeNet and ReLU-MLP [15]. It demonstrates that the MI values magnify the performance discrepancies between models as compared to mere accuracy scores shown in Figure 2b. In addition, the DenseNet is a better architecture for preserving useful information across layers/blocks. Furthermore, neither of the networks is guaranteed to converge by running 10,000 iterations that has been used as a default setting in many studies [9] as the MIs have a noticeable increase level when running 50,000 iterations.

### 5.2. CNN Kernel Analysis via MI Visualization

The information plane visualization reveals the striking evolution patterns of DL network layers during training and provides an objective indicator for the comparison of network convergence and performance. However, as shown in the Figure 4, the first couple of convolutional blocks of LeNet and DenseNet have high MI (the maximum MI value is log_2_ (10) = 3.32) when the MI between class label Y and kernels *M_i_* was estimated by concatenating the representations from all kernels of each block during training. Therefore, it is impossible to illustrate the evolution of the convolutional kernels, which are the basic units for extracting feature representations in most of the state-of-the-art DL networks. In our study, we further explore several other visualization techniques to illustrate the MI in the DL networks to better understand the kernel changes. The MI (Y; *M_i,k_*) is calculated between each convolutional kernel in the hidden layers and output label Y by setting *M_i,k_* as the response vectors from the k convolutional kernel of the *i* layer. The size of the representations extracted from each kernel of the first block in both CNN networks is 196 (14 × 14) and the size of the representations from each kernel of the second block of the networks is 49 (7 × 7). The Y label is set from 0 to 9 and the MI range is [0, log_2_ (10)] as it reflects how each kernel shares mutual information with its classification.

**Figure 5:**
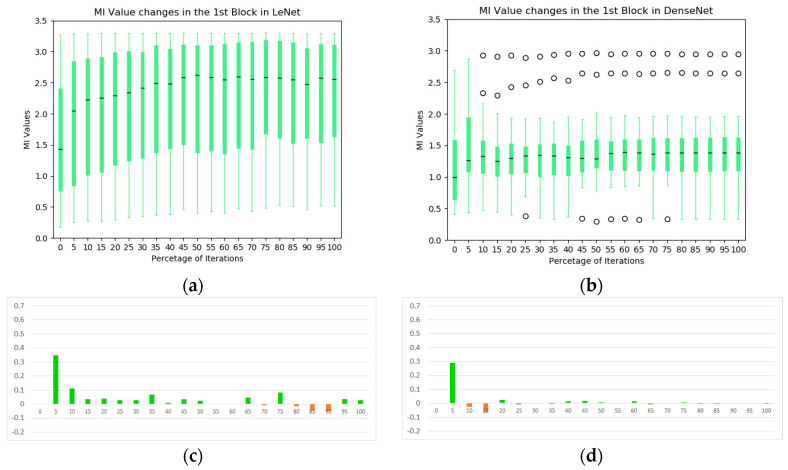
The statistics of MI between individual kernels and output labels during training: (**a**) the evolution of MI in the first convolutional block of LeNet; (**b**) the evolution of MI in the first convolutional block of DenseNet; (**c**) average MI change of the first block in LeNet; and (**d**) average MI change of the first block in DenseNet.

The box plot technique is one of the visualization techniques to analyze the MI statistics of convolutional kernels in the network. As illustrated in Figure 5, the black line in the middle of each box represents the median of the MI values between the extracted representations from individual kernels in first block and the output label. Here, the upper and lower boundaries of the box represent the first and third quartiles of the MI values in the block during training, and all values that are not in the range are considered outliers (open circles in the plots). The box plot summarizes the main characteristics of the data distribution and presents the outliers comparing to other types of statistics plot, e.g., the mean and standard deviation box plot. As a complementary analysis tool to the information plane in Figure 4, Figure 5 provides further insights into the convolutional blocks of the two CNNs. In the LeNet, the overall MI values of the individual kernels across the first convolutional block increase significantly at early stages of the training and they are higher than its counterparts in the DenseNet. In addition, we plot the first derivative of the average MI values at each iteration (Figure 5c,d). It is observed that the evolution of the DenseNet is more stable comparing to the LeNet. 

**Figure 6:**
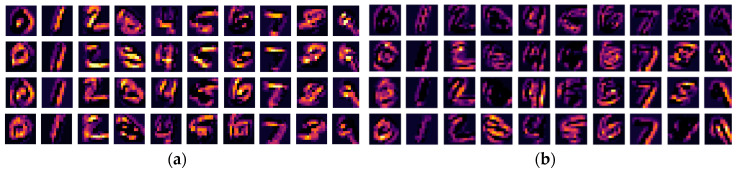
The MI guided LeNet activation maps after the first convolutional block: (**a**) the activation maps of the digits with the 4 kernels which have highest MI values; (**b**) the activation maps of the digits with the 4 kernels which have lowest MI values.

**Figure 7:**
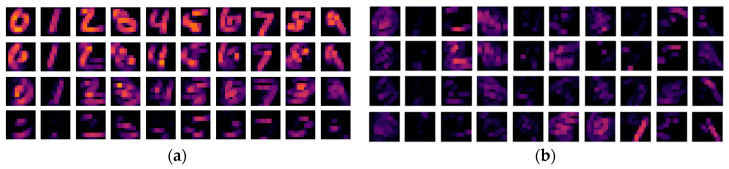
The MI guided DenseNet activation maps after the first convolutional block: (**a**) the activation maps of the digits with the 4 kernels which have highest MI values; (**b**) the activation maps of the digits with the 4 kernels which have lowest MI values.

The response maps generated from the first convolutional layer are more intuitive visual representations as they correspond to low-level visual features. We visualize the response maps by convolving the kernels that have the four highest and lowest MI values with a random sample in the test set to show the link between MI and feature representations. As illustrated in Figure 6 and Figure 7, both the kernels with the high MI values and those with the low MI values produce activation maps that are consistent with human perception to recognize the digits. Comparing between the LeNet and the DenseNet (Figure 6 vs. Figure 7), the response maps are blurrier in the DenseNet than in the LeNet. This is due to a larger convolutional stride used in the DenseNet, with which more information of the original image is lost in the operations. This is interesting because the final result is not sensitive to the convolutional stride in this block. These results confirm that some kernels are less effective in extracting distinctive information from the original images and create redundancies in the network.

**Figure 8:**
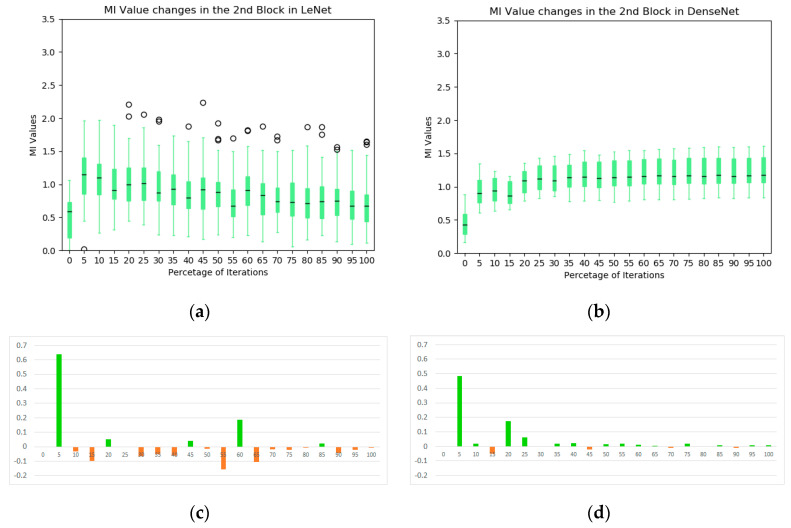
The statistics of MI between individual kernels and output labels during training: (**a**) the evolution of MI in the second convolutional block of LeNet; (**b**) the evolution of MI in the dense block of DenseNet; (**c**) average MI change of the second block in LeNet; and (**d**) average MI change of the second block in DenseNet.

The box plot of the MIs in the second blocks of the two networks are illustrated in Figure 8. In the LeNet, an increasing trend of MIs during the training process is not obvious, except in the very early stages of training, which is contrasted to the first block of the model (see Figure 5a). Indeed, the overall MI values tend to decrease toward the end of training. In the DenseNet, the overall MIs from the dense block of the DenseNet increase consistently and significantly (median value from 0.46 to 1.07). It indicates that the dense block learns representations that share more information with the final output. In addition, these kernels complement one another to keep high MI values for the following blocks (illustrated in the information plan visualization in Figure 4). From the average MI change plots (Figure 8c,d), it confirms the stableness during the evolution of the DenseNet.

### 5.3. Visualizing the Evolution of MI with Binary Label and MI Orders in CNNs

**Figure 9:**
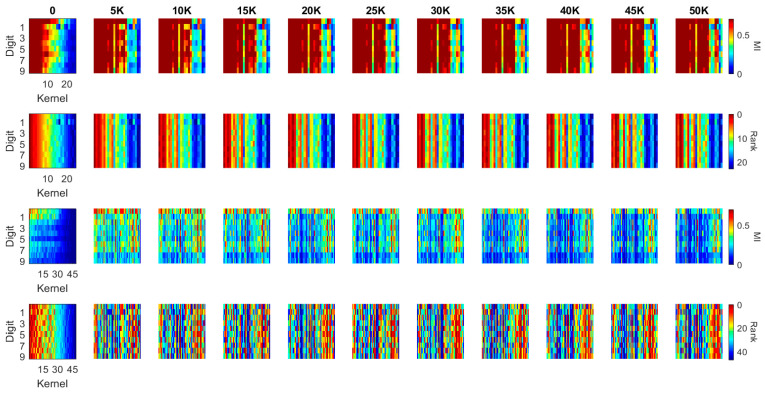
MI and MI order evolution of the kernels in the two convolutional blocks of LeNet. The kernels are in a descending order from left to right based on MI values at the initialization column. 1st row and 3rd row present the MI value evolution of the convolutional blocks while 2nd and 4th row present the MI order evolution of the corresponding blocks.

**Figure 10:**
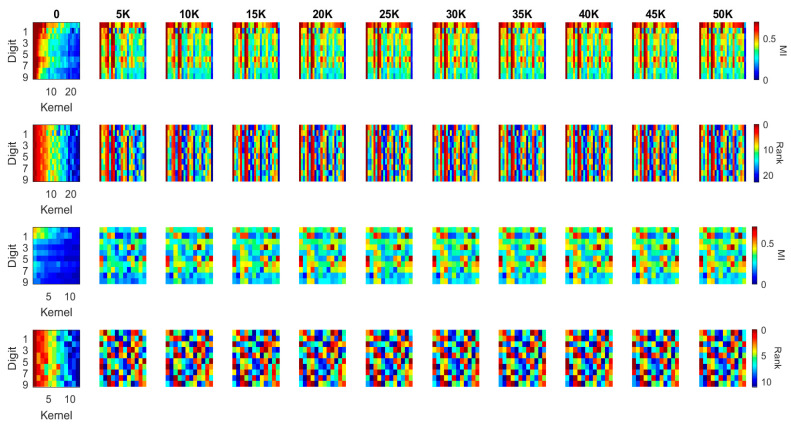
MI and MI order evolution of the kernels in the first two blocks of the DenseNet. The kernels are in a descending order from left to right based on MI values at the initialization column. 1st row and 3rd row present the MI value evolution while 2nd and 4th present the MI order evolution of the corresponding blocks.

The kernel MI calculated based on the 10 classes in the previous section illustrates the overall evolution pattern of the kernels in both the LeNet and the DenseNet. Another useful approach to calculate the MI values is setting the output (*Y*) to a binary flag to indicate how individual kernels contribute to the recognition of each digit (i.e., *Y* = 1 if the image contains such a digit; *Y* = 0 if the image contains another digit). In this case, the MI value between each kernel and the label ranges in [0, 1]. For each kernel in the first two blocks of the LeNet and the DenseNet, we calculated this MI value for each of the ten-digit classes (0–9) at every 5000 iterations during training (see Figure 9 and Figure 10). We used a heat-map, a popular visualization tool for identifying data patterns, e.g., [40,41,42], to visualize the evolution pattern for each kernel for each digit class. In these figures, each panel represents the “absolute” MI values (first and third rows) and the rank orders (second and fourth rows) of the kernels according to the “relative” MI values for a digit class. The absolute MI values for each digit class represents the correlation between a kernel activation and the model output when a given digit class is presented to the model, and a high correlation between a kernel and the model output indicates that the kernel activation represents some features of the digit class and contributes to the classification; a low correlation indicates that the kernel activation is less relevant to the eventual output and does not contribute to the classification much. The rank order (based on the relative MI values across different kernels in a given block) represents the degree to which a given kernel may contribute to the classification in relation to other kernels. By observing these values over iterations, it is possible to infer whether the kernels would become specialized to specific digit classes or features of digit classes over training. If the kernels would be specialized to a specific digit class or features of a digit class, then the absolute MI value would become higher for a digit class but lower for other digit classes over time. In a similar manner, the rank order of a kernel should change over time in such a way that the rank increases for a digit class but decreases for other digit classes over training. We applied this visualization techniques to individual kernels in the first two convolutional/dense blocks in the LeNet (see Figure 9) and the DenseNet (see Figure 10). In each panel, the horizontal axis represents individual kernels, and the vertical axis represents 10-digit classes (0–9).

As illustrated in Figure 9, the MI at the initial iteration in the first convolutional block (the first row) of the LeNet shows that some of the kernels consistently have high MI values for all digit classes, which means that these kernels preserve more distinctive information whereas other kernels with low MI kernels alter the input information in an inconsistent manner. During training, more kernels achieve higher MI values, which is consistent with the result in Figure 5a. An interesting observation is that when kernels have high MI values, they have high MI values for all digits, not for specific digits. This is also reflected in the rank order of the kernels (second row); kernels that are ranked higher are always ranked high across all digit classes, whereas those that are ranked lower are always ranked low across all digit classes. Therefore, the kernels are not trained to specialize for specific digits, but they are trained to achieve the consistency of information processing.

The second convolutional block of the LeNet, however, presents different patterns (see the third and fourth rows in Figure 9). From the initial iteration, there appears to be no kernel that produced high MI values (the third row), although the rank order still showed that some of the kernels have consistently higher MI values across all digits whereas others have low MI values (the fourth row). Over training, some kernels start producing high MI values, but there is a decreasing trend of MI values after 20,000 iterations, consistent with Figure 8b. The rank order also shows no clear pattern that some kernels have high MI values for all digit classes. Although the rank order shows such a trend toward the end of training in which one or two kernels consistently have high MI values across the digit classes, the pattern is not as obvious as that observed for the first convolutional block. Consequently, it can be said that the kernels in the second convolutional block are trained to specialize for particular characteristics of the digit classes.

In the first convolutional block of the DenseNet, there are fewer kernels with high MI values, as compared to the first convolutional block of the LeNet (see Figure 10, the first row), which is presumably due to the fact that the MI is generally lower in the first block of the DenseNet than in the first block of the LeNet (see Figure 5). Yet, the rank order (the second row) still shows that some kernels consistently have high MI values across all digit classes as in the first block of the LeNet. In contrast to the LeNet, however, the number of kernels that produce high MI values did not increase over training; instead, there appears to be more specialization of the kernels to particular digit classes, which are shown in both the MI values and the MI ranks. In the second block of the DenseNet, the general pattern is similar with that of the first block—a few kernels produce high MI values across all digit classes—but the kernels are more specialized for particular digit classes over iterations. These observations suggest that the DensNet is more efficient in learning specific features of the input images, as the MI order of the kernels changes more dramatically over iterations. In contrast, the update of the kernel weights in the first block of the LeNet is less efficient as the order of kernels does not change much over iterations. These results may reflect the possibility that the gradient descent method used to optimize the LeNet actually converges to a local maximum, whereas the DenseNet is more likely to find the global maxima. If so, the architecture with dense connections may be a better structure to reach DL solutions, which explains the better overall performance of the DenseNet.

In general, Figure 9 illustrates that the first convolutional block of LeNet has many high MI kernels that are able to generate efficient representations for all the 10 digits while the second convolutional block of LeNet has many low MI kernels. It indicates that there exist repetitive or similar kernels in the first block and many kernels in the second block have limited contribution to the task. The distribution of the MI values between the kernels and classes shows that the network is not a simplified architecture for the dataset. Comparing to the LeNet, Figure 10 illustrates a quite different pattern. Although the overall MI values in the first convolutional block of DenseNet are lower than the LeNet, it is able to generate a more compact representation in the second block which has a well distributed MI map (shown in the 3rd row of Figure 10). 

## 6. Further Analysis and Discussions

**Figure A1:**
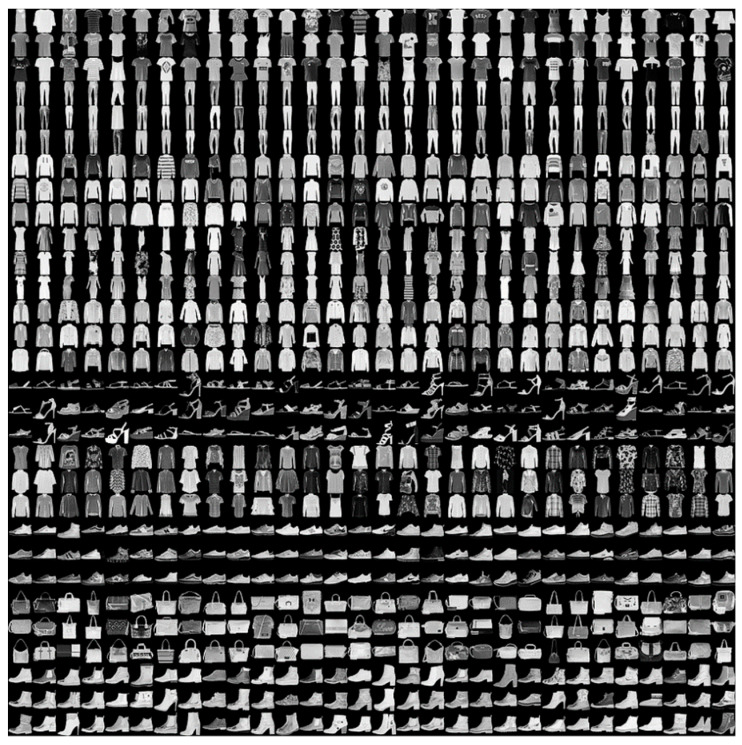
Fashion MNIST [43] is a new image dataset released in 2017 for benchmarking machine learning algorithms. It includes 10 classes, respectively named as T-Shirt, Trouser, Pullover, Dress, Coat, Sandals, Shirt, Sneaker, Bag and Ankle boots.

**Figure A7:**
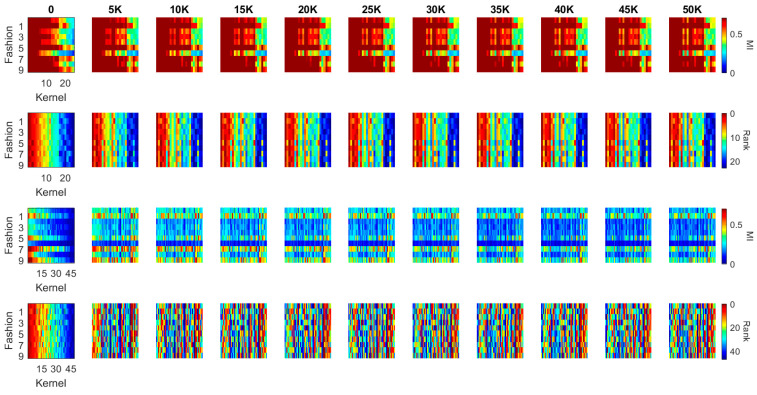
MI and MI order evolution of the kernels in the two convolutional blocks of LeNet. The X axis correspond to the kernels and the *Y* axis 0–9 correspond to T-Shirt, Trouser, Pullover, Dress, Coat, Sandals, Shirt, Sneaker, Bag and Ankle boots respectively. The kernels are in a descending order from left to right based on MI values at the initialization column. 1st row and 3rd row present the MI value evolution of the convolutional blocks while 2nd and 4th row present the MI order evolution of the corresponding blocks.

**Figure A8:**
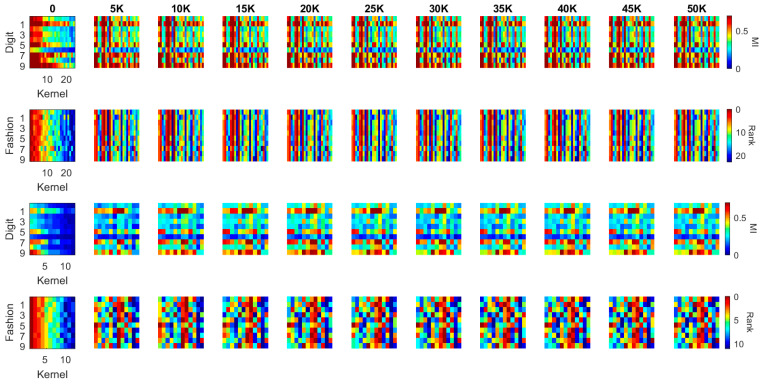
MI and MI order evolution of the kernels in the first two blocks of the DenseNet. The *X* axis correspond to the kernels and the *Y* axis 0–9 correspond to T-Shirt, Trouser, Pullover, Dress, Coat, Sandals, Shirt, Sneaker, Bag and Ankle boots respectively. The kernels are in a descending order from left to right based on MI values at the initialization column. 1st row and 3th row present the MI value evolution while 2nd and 4th present the MI order evolution of the corresponding blocks.

In addition to the MNIST dataset, we apply the visualization techniques on another popular dataset, Fashion MNIST [43] (See Appendix A). It is a new image dataset released in 2017 to benchmark machine learning algorithms, which includes 10 classes, named as T-Shirt, Trouser, Pullover, Dress, Coat, Sandals, Shirt, Sneaker, Bag and Ankle boots, respectively (illustrated in Figure A1). The applications of the visualization used in this present study provide useful insights into the networks based on the observations from the MI visualization. Our insights are able to not only facilitate the design of a more reliable, compact and accurate network architecture but also gain knowledge in the networks. The findings can be summarized as follows:

- In DL network design, it is assumed that a better network architecture is to preserve more useful information for classification and reduce the redundancy in the network. Illustrated in our work, MI, a theoretical measurement for quantifying the shared information between variables, can be used as an independent metric to evaluate the components in DL networks. Therefore, it is suggested that MI can be used in further research in two ways: (i) it can be used as an independent metric to evaluate new network architectures in addition to the accuracy rate; and (ii) it can be integrated into the optimization process for achieving better network performance.
- It is found that MI can be used to evaluate whether the network architectures are suitable for particular tasks. In specific, the MI becomes relatively stable and close to its IB bound on the information plane plots, for a dataset that has been well solved by DL methods (i.e., MNIST dataset). However, the MI values drop significantly during training when processing a more difficult dataset (i.e., Fashion-MNIST) and the convergence is farther from the IB bound.
- Based on the observation from Section 5.2 and Section 5.3 and figures in the Appendix A, it is found that there are different types of redundancy identified in the networks. For example, in the LeNet, highly correlated kernels in the first convolutional block generate the redundancy while less-effective kernels in the second convolutional block are the redundant components. It suggests that MI has the potential to guide the network developers to optimize the number of network kernels by identifying the redundancy in the layers. The purpose of using a large number of convolutional kernels at initialization step is to generate a more reliable feature extraction scheme although some kernel parameters may be stuck on local minima, making less contribution to the task. With the help of the MI, more efficient networks could be designed by removing those kernels for better optimization.
- From the visualization results in Section 5.2 and Section 5.3, it is easy to find that the convolutional layers are able to diversify the feature representations. More specifically, the heat-map visualization is a useful tool to identify the redundancy in DL networks by evaluating the MI distribution. In our study, it shows that the concatenation of the convolutional layers (the dense blocks in the DenseNet) can provide more distinctive features for classification. It facilitates a better combination of the blocks to achieve an improved architecture.
- Based on the observation from Figure 4, Figure 5, Figure 6 and Figure 7, we believe that larger stride setting in the first convolutional layer has little impact to the final performance when training on the MNIST data set. As shown in the Figure 5, the overall MI values from the kernels of Densenet are lower than the MIs in the LeNet. This has been visually evidenced by the illustrations in Figure 6 and Figure 7. In these two figures, the response maps of convolving 10 image instances with the 8 kernels (4 highest MIs and 4 lowest MIs) in the two networks. However, the MI evolution in the following layers (illustrated in Figure 4) shows that the final convergence is not influenced by the different settings in the two networks. Therefore, a larger stride in the network is a better choice to process the dataset.
- The following analysis is summarized from Figure 9 and Figure 10, Figure A7 and Figure A8: (1) when comparing the second block of the two networks, it is found that LeNet has many kernels, which generate low MI values with all the 10 classes (3rd row of Figure 9 and Figure A7). It indicates that the network is not efficient as there are many redundant kernels in the network. While MI between the kernels in the Densenet and the classes are distributed well across all the classes (3rd row of Figure 10 and Figure A8), which means each kernel is specialized to particular characteristics of classes; and (2) From Figure A7 and Figure A8, it is found that the MI in the fashion dataset corresponding to the fact that T-shirt, Pullover, Dress, Coat, and Shirt have relatively lower values comparing to the trouser, sandals, sneaker, bag, and boots classes (here shirt has the lowest MI). The observation is consistent on the second block of both the networks. This is understandable from the perception perspective as the 5 classes share similar visual stimuli comparing to the other classes. It suggests that a hierarchical network is preferable to achieve better performance comparing to a generic deep network.
- Based on the observation in Section 5.3, the evolution of kernels in the heat-map suggests that the gradient descent optimization becomes less effective for the early blocks once the network become deeper. As a result, it is useful to have either links from early blocks to the output layer or a better initialization to improve the optimization process. In contrast, based on the observation from the MI order evolution visualization, it is found that the dense blocks learn more compact representation as the redundancy in the features is less comparing to the convolutional blocks. It reflects that the concatenation of response map from a sequential of convolutional layers is a better learning strategy which can be further used in more network architectures.

## 7. Conclusions

In this paper, we attempted to bridge the gap between DL’s wide applications and its theoretical understanding via visualizing an information theoretic metric—MI. We (i) used three DL networks including ReluMLP, LeNet, and DenseNet; (ii) examined the evolution of MI during the training stage; and (iii) analyzed the response maps selected based on the MI in DL networks. Our work is indicative of the further use of MI in a number of ways. Practically, it can be (i) used as an independent metric to evaluate new network architectures; and (ii) integrated into the optimization process to achieve a better network performance. The use of MI could also enable the improvement of network architecture design by providing a better recognition of the role of each block in the networks and by removing redundant network components. We note that our observations are based on limited sets of networks; thus, further studies are needed to validate our work by evaluating via more datasets and network architectures. In practice, our findings can be used to guide the selection of suitable network architectures based on a particular task at hand and to evaluate the reliability of the new dataset for transfer learning. Furthermore, the findings from the analysis will aid to the simplification of the network, hyper parameters tuning as well as designing new optimization schemes.

In short, our current approach is most useful for investigating, tuning and selecting optimal network structure and hyperparameters that have a great potential to be used by model developers. In future, we plan to focus on using our techniques to help in practical applications and embedding the MI into various learning algorithms to automate the model building. Furthermore, our ultimate aim is to bridge the gap between DL’s theoretical understanding and its wider applications.

## Figures and Tables

**Figure A2:**
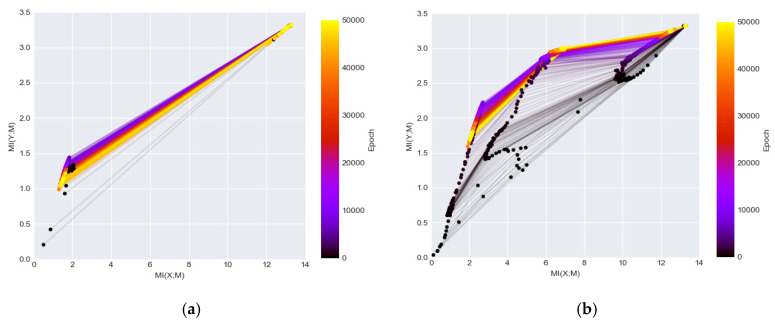
(**a**) The information plan visualization of LeNet on Fashion Dataset; and (**b**) the information plane visualization of DenseNet on Fashion Dataset. Comparing to the MNIST dataset, the Fashion is a harder task which converge to lower MI for both the networks.

**Figure A3:**
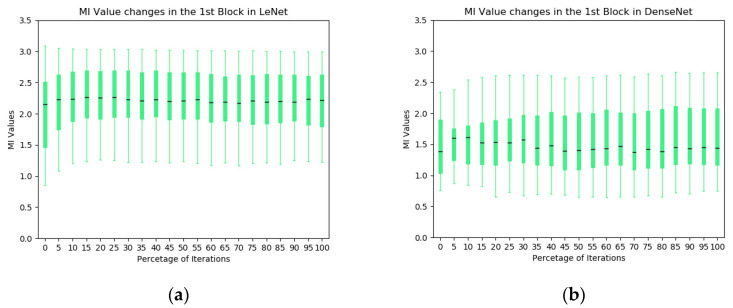
The statistics of MI between individual kernels and output labels: (**a**) the evolution of MI in the first convolutional block of LeNet; and (**b**) the evolution of MI in the first convolutional block of DenseNet. Comparing to the MNIST dataset, the MI values of kernels have insignificant increase indicating the learning is not efficient.

**Figure A4:**
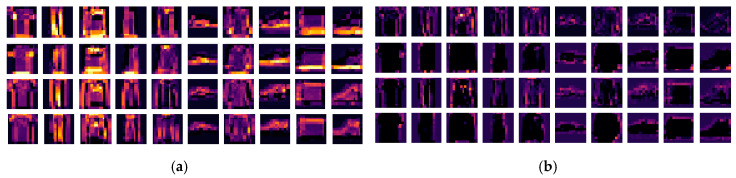
The MI guided LeNet activation maps after the first convolutional block on the Fashion dataset: (**a**) the activation maps of the digits with the 4 kernels which have highest MI values; (**b**) the activation maps of the digits with the 4 kernels which have lowest MI values.

**Figure A5:**
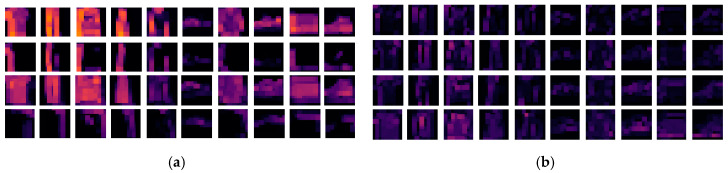
The MI guided DenseNet activation maps after the first convolutional block on the Fashion dataset: (**a**) the activation maps of the digits with the 4 kernels which have highest MI values; (**b**) the activation maps of the digits with the 4 kernels which have lowest MI values.

**Figure A6:**
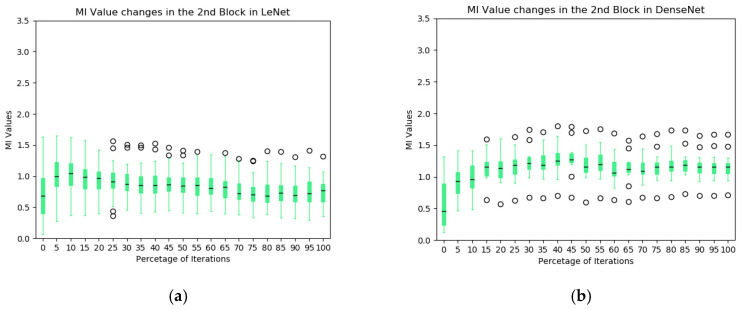
The statistics of MI between individual kernels and output labels: (**a**) the evolution of MI in the first convolutional block of LeNet; and (**b**) the evolution of MI in the first convolutional block of DenseNet. Comparing to the MNIST dataset, the MI values of kernels have insignificant increase indicating the learning is not efficient.
